# Subcortical Structures in Demented Schizophrenia Patients: A Comparative Study

**DOI:** 10.3390/biomedicines11010233

**Published:** 2023-01-16

**Authors:** Juan Rivas, Santiago Gutierrez-Gomez, Juliana Villanueva-Congote, Jose Libreros, Joan Albert Camprodon, María Trujillo

**Affiliations:** 1 Department of Psychiatry, Fundación Valle del Lili Cra. 98 # 18-49, Cali 760032, Colombia; 2 Department of Psychiatry, Universidad ICESI, Cali 760031, Colombia; 3 Department of Psychiatry, Universidad del Valle, Cali 760043, Colombia; 4 Hospital Departamental Psiquiátrico, Universitario del Valle, Cali 760035, Colombia; 5 Centre for Research and Training in Neurosurgery (CIEN), Bogotá 110411, Colombia; 6 Neurosurgery Department, Universidad de Nuestra Señora del Rosario, Bogotá 111711, Colombia; 7 Research Office, Hospital Universitario San Ignacio, Bogotá 110231, Colombia; 8 School of Systems and Computing Engineering, Universidad del Valle, Cali 760032, Colombia; 9 User-Centric Analysis of Multimedia Data Group, Technische Universität Ilmenau, 98693 Ilmenau, Germany; 10 Division of Neuropsychiatry, Massachusetts General Hospital, Harvard Medical School, Boston, MA 02129, USA

**Keywords:** schizophrenia, dementia, aged, hippocampus, amygdala, thalamus, neuropsychological tests

## Abstract

There are few studies on dementia and schizophrenia in older patients looking for structural differences. This paper aims to describe relation between cognitive performance and brain volumes in older schizophrenia patients. Twenty schizophrenic outpatients —10 without-dementia (SND), 10 with dementia (SD)— and fifteen healthy individuals —as the control group (CG)—, older than 50, were selected. Neuropsychological tests were used to examine cognitive domains. Brain volumes were calculated with magnetic resonance images. Cognitive performance was significantly better in CG than in schizophrenics. Cognitive performance was worst in SD than SND, except in semantic memory and visual attention. Hippocampal volumes showed significant differences between SD and CG, with predominance on the right side. Left thalamic volume was smaller in SD group than in SND. Structural differences were found in the hippocampus, amygdala, and thalamus; more evident in the amygdala and thalamus, which were mainly related to dementia. In conclusion, cognitive performance and structural changes allowed us to differentiate between schizophrenia patients and CG, with changes being more pronounced in SD than in SND. When comparing SND with SD, the functional alterations largely coincide, although sometimes in the opposite direction. Moreover, volume lost in the hippocampus, amygdala, and thalamus may be related to the possibility to develop dementia in schizophrenic patients.

## 1. Introduction

Schizophrenia is a severe mental disorder whose aetiology includes anatomical, genetic, and environmental factors, with significant effects on patients and society [1]. Cognitive disorders are a central finding in the disease [2], with some patients showing performance that is one standard deviation below the general population in cognitive testing. Nevertheless, in a significant number of patients, cognitive impairment is not described [3], suggesting that the problem is not clear yet.

There are different trajectories in the ageing processes of patients with schizophrenia compared to people with dementia and healthy individuals [4]. People with schizophrenia could have a higher risk of dementia than general population [4], but there is no consensus about these findings [5]. The risk for developing dementia is twice as high in patients with schizophrenia compared to healthy people, especially in those younger than 65-years [6,7,8,9,10].

Furthermore, the risk for dementia in late-onset and very late-onset schizophrenia can rise by 400% [11]. Inconsistencies among reports could be explained by multiple variables such as clinical status [3], severity of symptoms [12], number of hospitalizations [13], severity of negative symptoms [14], environmental factors [7], and somatic comorbidities [15]. Deficits in visuospatial orientation, memory, and attention have been described, even during the early stages of the disease [16]. However, there is no consensus on if abnormalities occur before patients are diagnosed with schizophrenia or if there is a cognitive decline that occurs across time [12]. 

In schizophrenia, the hippocampus is the structure that has been shown to have the largest volume loss [17,18,19]. Alterations are more frequently found on the left side, especially in the anterior hippocampus, CA1, and subiculum [20]. Although neuronal loss is not evident [21], a 4% bilateral hippocampal volume reduction can be observed regardless of the disease’s duration, age of onset, and medication. Patients with early-onset schizophrenia also show amygdala volume reduction on the left side, [17,19], in the basal nuclei, anterior amygdaloid area, paralaminar nuclei, and lateral nuclei [22]. Regarding the thalamus, the most frequent nuclei affected in schizophrenia are the dorsomedial (DM), ventral anterior (VA), and pulvinar, in which a reduction in the number and volume of neurons is evident [23]. Although the loss in thalamic volume seems nondependent on the chronicity of the disease [24], thalamic atrophy during the transition to psychosis in patients with poor prognosis has been reported [25].

Few studies focus on the risk of dementia in older schizophrenic patients and its relations with structural changes in the hippocampus, amygdala, and thalamus. Most of the studies evaluate young people in the first psychotic episode and they often ignore the effect of age on cognitive decline and structural changes [26,27,28,29,30]. Moreover, studies involving patients older than 50-years-old do not control for the risk factors inherent to the disease and those from the ageing process [10,26,31]. Additionally, there is scarce literature focused on the relations between schizophrenia and dementia in Latin American populations [32].

An understanding of structural deficits and cognitive impairment would allow us to develop biomarkers for older SZ and SZ dementia patients. The current study aims to investigate differences in cognitive performance, hippocampal, amygdala, and thalamic volume, and relations between cognitive functioning and structural changes in a group of schizophrenic patients older than 50 years.

## 2. Materials and Methods

### 2.1. Participants

Using a non-probabilistic sampling, we selected 20 outpatients and 15 healthy individuals, older than 50 years, from two hospitals in Cali, Colombia: Hospital Departamental Psiquiátrico Universitario del Valle (HDPUV) and Fundación Valle del Lili. We defined the groups as follows: 10 patients with schizophrenia without dementia (SND), 10 patients with the previous diagnosis of schizophrenia and recently diagnosed with dementia (SD), and 15 healthy subjects that were taken as the control group (CG). The symptoms of patients with schizophrenia started before 30 years old. The IRB of both hospitals approved the study and the Declaration of Helsinki was followed. All individuals signed informed consent before being included in the study.

To select subjects, we reviewed the charts of schizophrenic patients. In their clinical reports they were diagnosed at some point and were treated as such. JR reviewed every chart and two independent psychiatrists certified the diagnosis using DSM V criteria for schizophrenia. The Positive and Negative Syndrome Scale (PANSS) was used to evaluate the severity of positive and negative symptoms. Patients were on antipsychotics as part of their treatment. 

Dementia diagnosis was performed using the DSM V criteria for major neurocognitive disorders. Dementia patients belong to the neuropsychiatric clinic in HDPUV, and were evaluated by a neuropsychiatrist, neuropsychologist, and neuroradiologist. If there were no coincidences in diagnosis, an additional psychiatrist, with experience in dementia, was asked to perform the definitive diagnosis.

Exclusion criteria were neurological diseases such as epilepsy, stroke, traumatic brain injury, CNS infection, and brain tumours.

The null hypothesis was that there are no structural and functional differences when comparing the three groups. Raw data were previously published [33].

### 2.2. Cognitive Evaluation

Two neuropsychologists carried out screening, i.e., cognitive evaluations. They used MMSE and Addenbrooke’s Cognitive Examination (ACE). Additionally, the CDR and the Hachinski Ischemic Score (HIS) [34] were applied to confirm the presence of dementia. Finally, all subjects underwent the Yesavage Geriatric Depression Scale [35].

Neuropsychological tests included the Hopkins Verbal Learning Test (HVLT) [36], the Rey Complex Figure Test, [37], and the Free and Cued Selective Reminding Test (FCSRT) [38] for analysing memory. The Hopkins Verbal Learning Test and the FCSRT were applied for learning and memory capacity at the auditory–verbal level, and the Rey Complex Figure Test (RCFT) was used for the encoding and evocation of graphic visual material. The Boston Naming Test (BNT) was used for linguistic function [39].

The phonological fluency test (Letter F and S), [40] the semantic fluency test (animal creep), and the digit span test (DST) were used to assess prefrontal cortex functioning. Finally, the semantic fluency test (animal fluency) was also applied to assess mental flexibility and categorization.

### 2.3. Structural Reconstruction

All patients underwent MRI. Images were taken at the Fundación Valle del Lili using a 1.5 Tesla Siemens Avanto resonator, using the following parameters: repetition time (ms) 8000, echo time (ms) 99, inversion time (ms) 2371.2, flip angle 150, layer thickness 5 mm, space between layers 6 mm, voxel size 1 mm^3^, in axis x = 256, y = 256, and z = 0.898438.

### 2.4. Cortical Surface-Based Analysis

#### 2.4.1. FreeSurfer 6

Cortical reconstruction and volumetric segmentation were performed using the FreeSurfer 6 image analysis suite, which is available at http://surfer.nmr.mgh.harvard.edu/, accessed on 1 July 2019. The technical details of these procedures are described in several publications [41,42,43,44,45].

#### 2.4.2. Cortical and Volumetric Segmentation

This process includes: (1) motion correction and averaging of multiple volumetric T1 weighted images (when more than one is available), (2) removal of non-brain tissue using a hybrid watershed/surface deformation procedure [46], (3) automated Talairach transformation, (4) segmentation of subcortical white matter and deep grey matter volumetric structures—including the hippocampus, amygdala, caudate, putamen, ventricles—[42], (5) intensity normalization [47], (6) tessellation of the grey matter, the white matter boundary, automated topology correction [48], and (7) surface deformation following intensity gradients to optimally place the grey/white and grey/cerebrospinal fluid borders at the location with the most significant shift in intensity. This defines the transition to the other tissue class [41,49].

MRI images were automatically processed with the longitudinal stream in FreeSurfer 6 to extract reliable volume and thickness estimates [50]. Specifically, an unbiased within-subject template space image is created using robust, consistent inverse registration [50]. Several processing steps, such as skull stripping, Talairach transforms, MNI atlas registration, and spherical surface maps parcellations were initialised with shared information from the within-subject template for increased reliability and statistical power (Reuter et al., 2012).

For segmentation and parcellation of hippocampus and amygdala, we used the developing version at (https://surfer.nmr.mgh.harvard.edu/fswiki/HippocampalSubfields, accessed on 1 July 2019) with specific nomenclature [51]. For the thalamus, we used the developing version at (http://freesurfer.net/fswiki/ThalamicNuclei, accessed on 1 July 2019) with specific nomenclature [52].

#### 2.4.3. Statistical Analysis

Once data were obtained, we ran a normal distribution test on it and discovered that the data do not follow a normal distribution. Thus, we used non-parametric statistical tests. Differences between the three groups were tested using the Kruskal–Wallis and Mann–Whitney U tests, with neuropsychological scores and volumes of the thalamus, amygdala, and hippocampus. The null hypothesis was rejected with *p* ≤ 0.05. Regression models were used to explore relations between brain volumes and demographics data, along with cognitive performance, since there is no way to calculate correlation between three or more variables. The three demographic variables—age, years of schooling, mental disease duration—and neuropsychological scores were set as independent variables and a selected variable—volume of thalamus, amygdala, or hippocampus—was set as the dependent variable. The regression models were evaluated and selected based on the coefficient of determination, set at R^2^ ≥ 0.8. Once a regression model had R^2^ ≥ 0.8, the contribution of the independent variables to the model was assessed by the significance value of the coefficient of a variable—contributing to the model when *p* ≤ 0.05. We used the package Stata 16.0 (Stata Statistical Software StataCorp 2019) for the Kruskal–Wallis and the Mann–Whitney U tests, defining significance tests as *p* ≤ 0.05. Additionally, for each regression model, Spearman rank higher order correlation was used to corroborate pair relations between volume and demographic variables, as well as a neuropsychological test. We used ρ ≥ 0.8 for determining significance of the Spearman correlation.

Briefly, we used regression models for relating structure volumes with demographic and cognitive performances, then we corroborated those relations using Spearman correlations.

## 3. Results

We evaluated 35 individuals, 19 were women (54.2%). The median ages were 69.5 years for SD, 58 for SND, and 60 for CG. The Kruskal–Wallis tests showed significant differences between ages when comparing the three groups. Moreover, the Mann–Whitney U tests showed significant differences between ages of CG-SD and SD-SND (*p* 0.006 and 0.003 respectively), but not when comparing CG to SND (*p* = 0.18). The schizophrenic groups had a lower educational level than CG, without finding significant differences between them (*p* = 0.21). There was a significant difference in mental illness duration (*p* = 0.0042), with a median of 41.3 years in the SD group and 26.9 in the SND. Table 1 shows the order statistics of demographic variables, along with the obtained Mann–Whitney U tests *p*-values of comparison between groups.

In the PANSS scale, there were significant differences between the groups in negative symptoms and general psychopathology, with better performance in SND. Patients had difficulty in abstract thinking (*p* = 0.015), stereotyped thinking (*p* = 0.039), anxiety (*p* = 0.034), uncooperativeness (*p* = 0.009), disorientation (*p* = 0.001), poor attention (*p* = 0.001), lack of judgment and insight (*p* = 0.001), disturbance of volition (*p* = 0.008), poor impulse control (*p* = 0.016), and in the total general psychopathology scale (*p* = 0.001). There were no significant differences in positive symptoms. Order statistics of test results are detailed in Appendix A (Table A1).

Patients were on antipsychotics as part of their treatment. Throughout the course of their illness, they received both typical and atypical antipsychotics. Since SD were older, obviously they were exposed for longer periods of time to pharmacological management than SND. However, the registers are of formulation, but there is no certainty of adherence to it.

Table 2 summarises the quartiles of score of the different neuropsychological tests for the functional analysis and the obtained *p*-values of Mann–Whitey U tests. There were significant differences in overall cognitive condition between the three groups. We found that the CG performed within normal limits, while patients with schizophrenia from both groups had an inferior performance, with evident alterations in the different domains. Moreover, we observed a greater severity in SD than in SND in all tests, except the semantic memory and visual attention tests.

### 3.1. Structural Analysis

**Figure 1:**
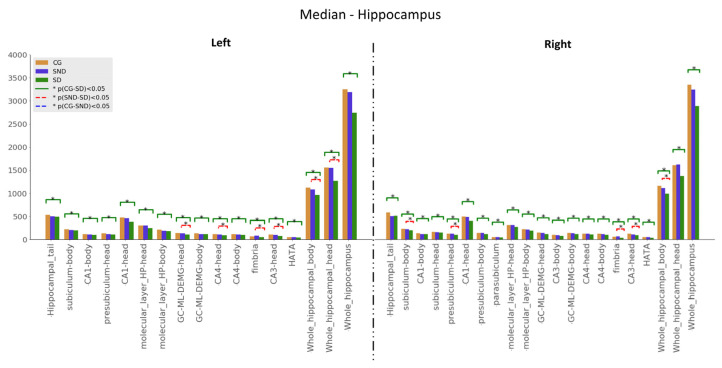
Comparison of medians, $H_{0}:\mu_{i}=\mu_{j}$, with Mann–Whitney U test, with * *p* ≤ 0.05, for hippocampus structures.

**Figure 2:**
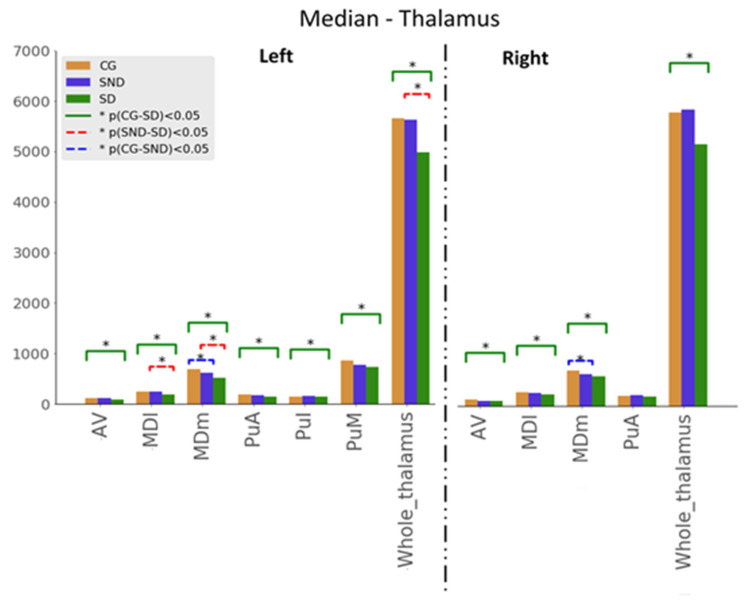
Comparison of medians, $H_{0}:\mu_{i}=\mu_{j}$, with the Mann–Whitney U test, with * *p* ≤ 0.05, for thalamus structures (AV: Anteroventral, MDm: Mediodorsal medial, MDl: mediodorsal lateral, PuA: Anterior pulvinar, PuI: Inferior pulvinar, PuM: Medial pulvinar).

**Figure 3:**
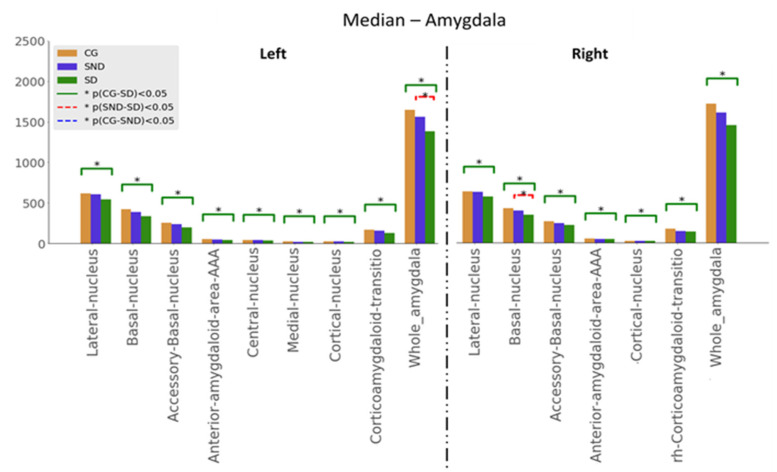
Comparison of medians, $H_{0}:\mu_{i}=\mu_{j}$, with the Mann–Whitney U test, with * *p* ≤ 0.05, for amygdala structures.

Kuskal–Wallis tests were used for examining the differences between the three groups for the set of variables. Mann–Whitney U tests results—with *p* ≤ 0.05—are illustrated in Figure 1, Figure 2 and Figure 3. The green line indicates differences between CG and SD, the red line indicates differences between SD and SND, and the blue line indicates differences between CG and SND. Moreover, the *y*-axis represents the magnitude of volumes and the *x*-axis corresponds to the segmented structures. 

#### 3.1.1. Hippocampus

Hippocampal volumes did not show significant differences when comparing SND with CG using Mann–Whitney U (See Figure 1). When we compared SD with CG, there were significant differences in every segment, with higher compromises on the right side. On the left side, SD had less volume than CG at the granular layer in the head of the dentate gyrus, head of CA4, fimbria, head of CA3, body, and head of the hippocampus. On the right side, findings reached significance on the head and body presubiculum, fimbria, head of CA3, and the entire hippocampus’s body. Medians are presented in Appendix B (Table A2).

#### 3.1.2. Thalamus

In the thalamus analysis, we used the non-motor or sensory relay nuclei: the anterior (AV), the dorsal medial (MDI), and the pulvinar. The latter was subdivided into subnuclei, namely anterior (PuA), lateral (Pul), medial (PuM), and posterior. When comparing SD with CG, all the volumes were significantly higher in CG with statistically significant results expected in the right PuA (Figure 2). Comparisons between SND and SD showed statistically significant results in the left MD nucleus (both parvocellular and magnocellular divisions) and the left thalamus’s whole volume, higher in SND than SD. In all structures under investigation, volumes were higher in CG > SND > SD, except in the right thalamic whole volume, in which SND held the higher measurements followed by CG and SD. Medians are presented in Appendix B (Table A3).

#### 3.1.3. Amygdala

Amygdala analyses were based on the anatomical nuclear division, as shown in Figure 3. As in all the previous analyses, comparisons between CG and SD showed significant differences in all the studied structures, with SD having the lowest volumes. Regarding SND vs. SD comparisons, statistical significance was reached for the left whole amygdala volume and in the right basal nucleus, with the lowest volumes in SD. In all structures, volumes were ordered as follows: CG > SND > SD. Overall, the left amygdala showed lower volumes than the right amygdala. Medians are presented in Appendix B (Table A4).

### 3.2. Structure vs. Function Analysis

Linear regression models were calculated to establish relations between the volumetric variables—each as the independent variable—and demographic variables, along with neuropsychological test scores as dependent variables. If R^2^ ≥ 0.8, the regression model is considered representative of data and selected as a good model for representing relations between volumetric and significant dependent variables.

**Figure 4:**
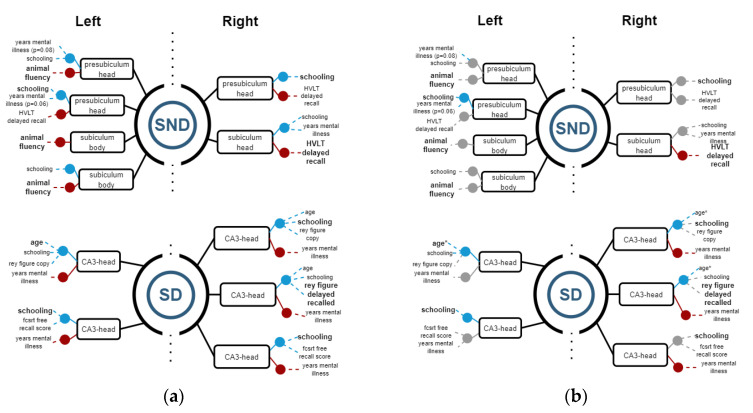
(**a**) Regression models with R^2^ ≥ 0.8, indicating the structure variables in a black rectangle aligned with blue (+) and red (−) points for the significant independent variables. (**b**) Spearman correlations with ρ ≥ 0.8 for corroborating relations between the structures and demographic and cognitive variables. Grey points means that there is no correlation.

**Figure 5:**
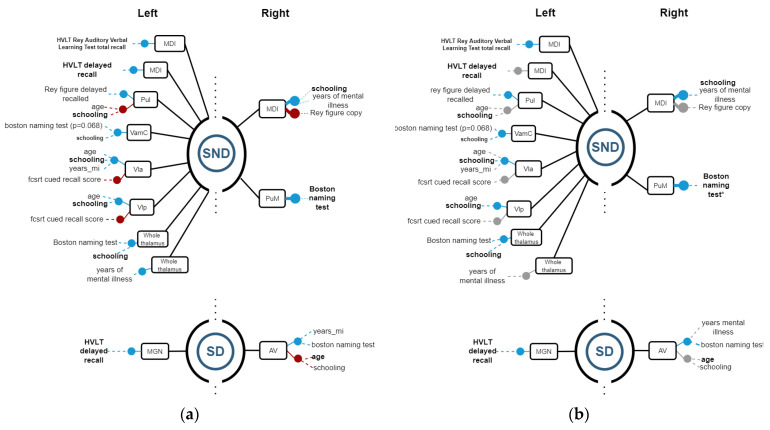
(**a**) Regression models with R^2^ ≥ 0.8, indicating the structure variables in a black rectangle aligned with blue (+) and red (−) points for the significant independent variables. The graph on (**b**) indicates Spearman correlations with ρ ≥ 0.8 for corroborating relations between the structures and demographic and cognitive variables. Grey points mean that there is no correlation.

**Figure 6:**
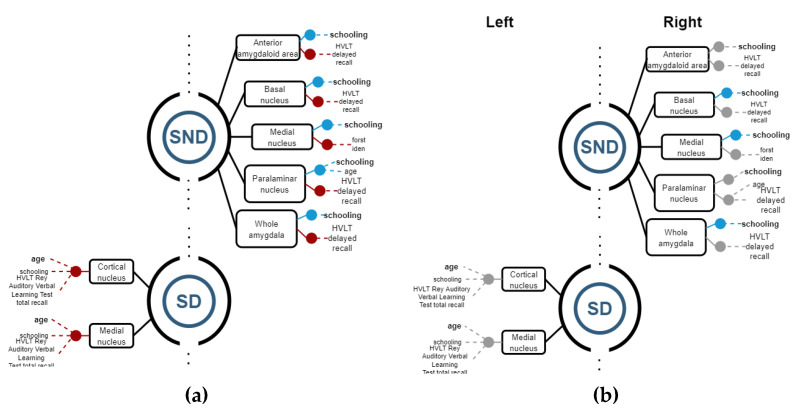
(**a**) Regression models with R^2^ ≥ 0.8, indicating the structure variables in a black rectangle aligned with blue (+) and red (−) points for the significant independent variables. (**b**) Spearman correlations with ρ ≥ 0.8 for corroborating relations among the structures and demographic and cognitive variables. Grey points means that there is no correlation.

Then, Spearman correlations were calculated for corroborating possible relations between structures and functions. Figure 4, Figure 5 and Figure 6 show regression models with R^2^ ≥ 0.8, where black boxes correspond to the structures, blue lines indicate positive relations, and red lines, negative ones with cognitive performance and demographic data that each variable coefficient contributed to the model (*p* ≤ 0.05). Additionally, Spearman correlation results are shown in Figure 4, Figure 5 and Figure 6, where grey points mean that there is no correlation.

The regression models for the CG did not yield R^2^ ≥ 0.8. The larger R^2^ values on the right side were 0.36 in the hippocampus, 0.56 in the thalamus, and 0.31 in the amygdala, while on the left side, they were 0.62 in the hippocampus, 0.58 in the thalamus, and 0.23 in the amygdala.

#### 3.2.1. Hippocampus

In SND patients, the left head presubiculum showed a positive correlation with schooling and a negative correlation with animal fluency and HVLT delayed recall. Similarly, the subiculum body showed a positive correlation with schooling and a negative correlation with animal fluency. The right side analyses showed relations in the head presubiculum and subiculum, in such a way, that the presubiculum was positively related to schooling and negatively to HVLT delayed recall, whereas the subiculum head had a positive relation with schooling and disease years, while showing a negative relation to HVLT delayed recall test (Figure 4).

On the other hand, SD patients showed relations in the CA3 segment of the hippocampal head. On the right side, positive relations were found regarding age, schooling, Rey complex figure copy, FCSFT free recall score, and a negative relation regarding years of the disease. On the left side, positive relations were found with age, schooling RCFC and delayed recall, and FCSRT free recall score; negative relations were related to years of diagnosis (Figure 4).

In SND, only HVLT showed a negative correlation with the right subiculum head, while in the left side, only schooling had a positive one. In the SD group, there were more correlations: CA3 head on the right side had positive correlation with age, and negative ones with years of mental disease. On the left side, there were positive correlations between head of presubiculum and schooling, and a positive correlation between CA3 head, age, and schooling.

#### 3.2.2. Thalamus

Among SND patients, regression models showed relations on the left side similar to their SD counterparts. On the left side, the MDl had a positive relation with HVLT total recall and the MDl had a positive relation with HVLT delayed recall. The Pul volume can be estimated based on a positive relation with the Rey figure delayed recall and negative relation with age and schooling. The magnocellular portion of the AV nucleus was positively related to schooling and animal fluency. The VLA had positive results with age, years of mental disease, and schooling, while showing a negative relation with cued recall score. The whole left thalamus had positively related results in schooling, Boston naming tests, and years of the disease. On the right side thalamus in the SND group, the MDl nucleus had positive relations with schooling and years of disease, while showing negative relations with Rey’s figure copy. The Pul had a positive correlation with years of the disease. The PuM nucleus had a positive relation with the Boston naming test and years of mental disease (Figure 5).

In SD patients, relations were found on the right side. The right AV nucleus was negatively related to digit span, age, schooling, and years of diagnosis. Additionally, the AV nucleus was positively related to years of disease and the Boston naming test. On the left side, MGN had a positive relation with HVLT delayed recall (Figure 5).

Regarding Spearman correlations, we found positive correlations on the right side between MCI and years of mental disease, along with PuM and Boston naming in SND. On the left side, there were positive correlations between MCI and total recall of HVLT Rey’s words, Pul and Rey´s figure delayed recall, VamC and schooling, Vla and schooling, Vlp and schooling, and the whole thalamus and Boston naming. In SD, on the right side, AV was correlated with years of mental disease, while on the left side, MGN had a positive correlation with hvlt delayed recall.

#### 3.2.3. Amygdala

In SND patients, all statistically significant results between structures and functions were found on the right side. All the right segments had a strong positive relation to schooling, while most of them showed negative correlations with the HVLT delayed recall test except the medial nucleus, where a negative relation was found on the FCRST Iden Test. The right paralaminar nucleus also showed a positive correlation with age but to a lesser extent with schooling. Regarding SD patients, only negative relations were found, and only on the left side. The medial and the cortical nucleus had negative relations with age (mainly), schooling, and HVLT words of Rey total Recall tests (Figure 6).

By the correlation analysis, we corroborated, in SND on the right side, that basal nuclei, medial nuclei, and whole amygdala had a positive correlation with schooling. In SD, significant correlations were not possible to corroborate on the left side.

## 4. Discussion

We evaluated 35 individuals to compare cognitive performance and brain volumes. Cognitive performance was significantly lower in schizophrenia patients (SD and SND) than in CG. Structurally, we found statistically significant differences among schizophrenia patients when compared to CG in the hippocampus.

The cause of different performance in cognitive evaluation remains unclear. Demographic and other non-schizophrenia-related factors such as age did not fully explain this finding. If age was indeed a factor, SND patient performance would have been similar to CG and not to SD. Both groups of patients with schizophrenia presented failures in the same cognitive domains. While overall cognitive impairment was greatest in SD over SND, visuo-graphic memory and executive system function impairment was consistent between the two schizophrenia groups.

Although it is well known that schizophrenia causes a degree of cognitive impairment and that cognitive deficits in these patients are established early in the disease, which makes them distinguishable from the healthy population. The structural basis for its onset appears to be different from that found in AD or frontotemporal dementia (FTD) [8,25,31,53,54,55,56,57,58,59,60,61,62,63,64,65,66,67,68,69,70,71].

Deficits in left CA1, subiculum, and DG were shown in schizophrenia [20,72], while in AD, there is a compromise of CA1 and the subiculum, preserving CA3 and DG [73]. There was a gradual reduction in the volume of CA3 in the SD in our sample, directly related to visual memory. The latter could imply a coexistence of two pathologies, explained by factors such as the sample size, the presence of dementia other than AD, or the chronicity of schizophrenia which further deteriorates these structures. An analogous situation occurred with the head of the left subiculum, which had a negative relation with audio-verbal memory in SD, but not in SND. This alteration could be caused by the dementia process and not for schizophrenia; therefore, CA3 volume and its associated function may also constitute markers of cognitive impairment in schizophrenic patients.

In the thalamus, the magnocellular portion of the left MD nucleus was significantly smaller in SD than SND and CG and SND than CG, suggesting that schizophrenia could be the preponderant factor for reducing the size of this nucleus. On the right side, the differences are significant when comparing the groups with schizophrenia to the CG, but not when comparing SD with SND. This thalamic alteration could be a specific compromise for the dementia process, and not influenced by the schizophrenic process.

The thalamic nuclei associated with cognitive functions are the DM, AV, and pulvinar [74,75,76]. Consequently, alterations in these structures have been associated with cognitive deficits, such as attentional, executive, and language failures in schizophrenia [77,78]. Thalamic alterations in our sample were evident in the nuclei related to cognition on the left side and related, although secondarily, to short-term memory processes.

Regarding the amygdala, there were no significant differences between CG and SND, and a few regions with significant differences between SD and SND, which led us to think that the loss of volume in this structure is not associated with schizophrenia and that the associated mental symptoms may be a consequence of its atrophy. This may imply that, unlike AD, where the main alterations occur in the hippocampus, in schizophrenia, a predominant factor in the genesis of dementia is the atrophy of specific nuclei of the amygdala and thalamus. A structural marker of risk or early diagnosis of dementia in schizophrenic patients could therefore be available, since there is evidence of the modulation that the amygdala exerts on the hippocampus for episodic memory [79,80,81].

The Spearman correlation and the linear regression did not always show relations between structural differences and cognitive function. This implies that the cognitive function does not rely only on volumetric measurements of brain structures, but on the connectome integrity in terms of functional analysis. It will be necessary to consider that volume does not necessarily relate to function or that there are compensatory brain mechanisms against the atrophy of some of its structures.

The lack of coincidence between the findings previously reported in the literature and our results can be explained by the analysis and segmentation methods, and the age group. Segmentation of the brain is a complex procedure and only through sophisticated image processing methods is it possible to separate some structures from others. The used FreeSurfer version 6 allows a more precise segmentation of small structures, such as the specific thalamic nuclei and the amygdala, and differentiation of segments of the hippocampus.

## 5. Conclusions

Cognitive performance and structural changes allowed us to differentiate between schizophrenia patients and CG. Changes in both domains were more evident in SD than in SND, suggesting that schizophrenia may be a risk factor for developing dementia symptoms and that may be explained by changes in the hippocampus, thalamus, and amygdala.

When comparing SND with SD, the cognitive alterations coincide, although sometimes in the opposite direction. This could be explained in several ways: (a) an insufficient sample size, (b) the impact of demographic variables, especially age, and (c) the duration of the mental illness. Other structures than expected for the underlying pathology appear to be compromised. If this is the case, these differentiated structures may become markers of deterioration for patients with schizophrenia and without dementia.

This study has several strengths and limitations. Our research has various novelties. First, we selected a cohort of patients older than 50, which has not been frequently reported in the literature. Second, we used image segmentation software (FreeSurfer-6), which allowed us to obtain more details than previously published studies. Additional, there are few studies on the relation between schizophrenia and dementia in Latin America [32]. Finally, we did a structure–function analysis looking for possible relations between them.

Among the limitations, the first one is the sample size, a drawback that arises from several facts. The prevalence of dementia in schizophrenic patients is not established; therefore, an exploratory study was conducted. The obtained results cannot be extrapolated to patients universally. Moreover, there were economic limitations that prevented a larger sample size and deepened possible causal factors of dementia symptoms. In the findings, the age bias shown by the SD group implies that age may be a confounding factor. This inconvenience arose due to the limited number of patients that were enrolled.

## Figures and Tables

**Table 1:** Demographic variables. CG: Control Group; SND: Schizophrenia without Dementia; SD: Schizophrenia with Dementia.

	CG Q2 (Q1–Q3)	SND Q2 (Q1–Q3)	SD Q2 (Q1–Q3)	Mann–Whitney *p*-Value		
				CG vs. SND	CG vs. SD	SD vs. SND
Age (years)	60.0 (56.0–64.0)	58.0 (52.0–59.8)	69.5 (66.5–72.5)	0.1810	0.0060	0.0030
Schooling: completed years	15.6 (11.0–22.0)	8.9 (5.0–14.0)	6.8 (0–15.0)	0.0001	0.0001	0.2122
Years of schizophrenia	-	26.9 (15.0–45.0)	41.3 (27.0–55.0)	-	-	0.0042

**Table 2:** Quartiles of neuropsychological scores. CG: Control Group; SND: Schizophrenia without Dementia; SD: Schizophrenia with Dementia.

	CG Q2 (Q1–Q3)	SND Q2 (Q1–Q3)	SD Q2 (Q1–Q3)	Mann–Whitney *p*-Value		
				CG vs. SND	CG vs. SD	SD vs. SND
LetterF	6 (4–7)	4 (3.8–5.3)	0 (0–2.3)	0.039	0.001	0.001
Animal Fluency	7 (6–7)	3 (1.8–4.3)	0 (0–2)	0.001	0.001	0.005
LetterS	6 (4–7)	3 (0–4)	0 (0–0.3)	0.002	0.001	0.034
HVLT Rey words-Total Recall	105 (87–116)	56.5 (41–75)	25.5 (14.3–42.3)	0.001	0.001	0.009
HVLT-Delayed Recall	11 (9–14)	4.5 (3.8–7.3)	0 (0–3)	0.001	0.001	0.007
Rey Figure-Copy	35 (32–36)	25.5 (18.4–32)	7 (0–14)	0.003	0.001	0.025
Rey Figure-Immediate Recall	18 (14.5–28)	7.5 (0–11)	0 (0–3.8)	0.001	0.001	0.122
Rey Figure-Delayed Recalled	18 (14–22.5)	7.5 (0.4–13.5)	0 (0–2)	0.001	0.001	0.024
Digit Span	5 (4–7)	4 (2.8–4)	2.5 (1.5–3.0)	0.005	0.001	0.012
Boston Naming Test	19 (19–20)	8 (0–19.3)	11.5 (0–15)	0.018	0.001	0.617
FCSRT-IDEN	16 (16–16)	15 (13.8–16)	12.5 (7.5–15.3)	0.001	0.001	0.077
FCSRT-Free Recall score	35 (31–40)	18 (12.8–26.5)	8.5 (0–13.5)	0.004	0.001	0.022
FCSRT-Cued Recall score	43 (40–46)	24 (17.3–30.8)	7 (0–21.3)	0.001	0.001	0.033
FCSRT-Total recall score	73 (55–84)	46.5 (37.5–55.5)	18 (0–4)	0.017	0.001	0.015

## Data Availability

All the original data used in this research are available at https://zenodo.org/record/3901876, accessed on 20 June 2020 [82].

## Appendix A

**Table A1:** Order statistics of PANSS scores.

Variable	SND Q2 (Q1–Q3)	SD Q2 (Q1–Q3)	*p*-Value
P1 Delusion	4.5 (2.75–5)	3.5 (3–5.25)	1.000
P2 Conceptual disorganisation	3.5 (2.75–4.25)	4.5 (3.75–5)	0.078
P3 Hallucinatory behaviour	2.5 (2–4)	2.5 (2–4.25)	0.522
P4 Excitement	2 (2–2.25)	3 (2–3.25)	0.072
P5 Grandiosity	2.5 (2–3.25)	3 (2–3.25)	0.684
P6 Suspiciousness/persecution	4 (3–4)	4 (2–5)	0.905
P7 Hostility	2 (1.75–3)	2 (2–4)	0.245
N1 Blunted affect	5 (3.75–6)	4 (3.75–6)	0.580
N2 Emotional withdrawal	5 (3.75–6)	5 (3.75–6)	0.937
N3 Poor rapport	5 (3.5–6)	5 (3.75–6)	0.724
N4 Passive/apathetic socialwithdrawal	5 (3.5–5.25)	5 (4–5.25)	0.691
N5 Difficulty in abstract thinking	5 (4.5–5)	6 (5.5–6)	0.015
N6 Lack of spontaneity andflow of conversation	5 (3.75–6)	6 (4.75–6)	0.094
N7 Stereotyped thinking	5 (3.75–5)	6 (4.75–6)	0.039
G1 Somatic concern	4 (3–4.25)	5 (4–6)	0.056
G2 Anxiety	3 (2–4)	4 (3.75–5)	0.034
G3 Guilt feelings	2 (1.75–2)	2 (2–3)	0.061
G4 Tension	3 (2–4.25)	3 (2–4)	1.000
G5 Mannerisms and posturing	3 (2–3)	2 (2–3)	0.622
G6 Depression	2 (2–3)	2.5 (2–4)	0.270
G7 Motor retardation	3.5 (2–4)	3 (2–4)	0.872
G8 Uncooperativeness	3 (2–4)	5 (4–6)	0.009
G9 Unusual thought content	5 (2.75–5)	5.5 (3.75–6)	0.138
G10 Disorientation	2 (2–2)	6 (5.75–6)	0.001
G 11 Poor attention	2 (2–3)	6 (5–6)	0.001
G12 Lack of judgement and insight	4 (3.75–5)	6 (5–6)	0.001
G13 Disturbance of volition	4.5 (3.75–5.25)	6 (5.75–6)	0.008
G14 Poor impulse control	2.5 (2–3.25)	5 (3.5–5.25)	0.016
G15 Preoccupation	2.5 (2–4.25)	4 (3.5–5.25)	0.072
G16 Active social avoidance	5 (3–5.25)	6 (4.75–6)	0.068
Total P—PANSS	21 (16.75–24.25)	22.5 (17.75–29.25)	0.343
Total N—PANSS	35 (27.25–38.5)	36.5 (32.5–39.5)	0.472
Total G—PANSS	53 (42–55.75)	67 (64.75–77.5)	0.001

## Appendix B

**Table A2:** Medians of substructures from Hippocampus (lh: left side, rh: right side).

Variable	CG	SND	SD
lh-Hippocampal_tail	533.1768	502.179	491.2918
lh-subiculum-body	221.7534	209.4655	198.4704
lh-CA1-body	118.8101	108.485	102.6303
lh-subiculum-head	184.9953	178.9422	165.2528
lh-hippocampal-fissure	138.3868	137.3062	155.3961
lh-presubiculum-head	134.1008	120.8248	109.1787
lh-CA1-head	474.0209	460.1142	384.2375
lh-presubiculum-body	153.4684	147.7923	125.0648
lh-parasubiculum	56.16448	50.60878	51.01351
lh-molecular_layer_HP-head	301.2178	305.4716	247.7981
lh-molecular_layer_HP-body	213.2131	192.8378	181.2546
lh-GC-ML-DEMG-head	138.8135	131.2461	111.1553
lh-CA3-body	83.18301	75.73118	77.4677
lh-GC-ML-DEMG-body	129.4957	116.4267	113.3807
lh-CA4-head	116.6823	110.3765	92.76147
lh-CA4-body	116.1934	106.2959	103.3087
lh-fimbria	66.43488	81.87439	50.07478
lh-CA3-head	109.5836	99.03824	75.07533
lh-HATA	52.55361	53.39614	41.60395
lh-Whole_hippocampal_body	1122.199	1083.858	966.5157
lh-Whole_hippocampal_head	1553.4	1545.423	1272.626
lh-Whole_hippocampus	3250.59	3191.603	2744.368
rh-Hippocampal_tail	580.5496	505.6549	510.067
rh-subiculum-body	231.9206	227.1127	199.9714
rh-CA1-body	134.9102	115.9702	116.1926
rh-subiculum-head	168.9085	162.5255	153.7425
rh-hippocampal-fissure	156.8318	145.9764	174.7104
rh-presubiculum-head	124.5519	122.7295	103.8624
rh-CA1-head	497.8928	489.8866	406.8071
rh-presubiculum-body	142.5037	138.6225	119.0961
rh-parasubiculum	55.66079	54.70945	43.47544
rh-molecular_layer_HP-head	317.6802	313.378	270.7611
rh-molecular_layer_HP-body	226.9824	213.1586	187.8682
rh-GC-ML-DEMG-head	150.4251	145.2717	119.7443
rh-CA3-body	98.27856	96.03128	78.87846
rh-GC-ML-DEMG-body	138.4234	133.6741	113.8646
rh-CA4-head	124.3385	122.6584	106.5683
rh-CA4-body	122.7553	117.8695	103.7635
rh-fimbria	64.06755	65.34351	38.52792
rh-CA3-head	122.4057	107.8549	94.62282
rh-HATA	54.12791	52.05819	37.70217
rh-Whole_hippocampal_body	1157.326	1109.135	985.3122
rh-Whole_hippocampal_head	1600.391	1616.814	1371.619
rh-Whole_hippocampus	3334.209	3221.467	2877.293

**Table A3:** Medians of substructures from Thalamus (lh: left side, rh: right side).

Variable	CG Q2	SND Q2	SD Q2
lf-AV	123.2183	113.6766	95.21425
lf-CeM	58.49963	54.05158	44.02321
lf-CL	32.81186	30.63348	27.25588
lf-CM	224.751	256.3122	223.2634
lf-LD	25.71389	25.69633	16.54721
lf-LGN	155.1749	142.4712	114.0722
lf-LP	113.6153	110.5227	99.43813
lf-L-Sg	17.44105	22.1667	24.09774
lf-MDl	252.15	241.727	193.1979
lf-MDm	685.4231	613.685	511.9704
lf-MGN	110.7312	96.8326	92.98687
lf-MV(Re)	11.19719	9.730469	8.056666
lf-Pc	3.213006	3.061099	2.771158
lf-Pf	50.18333	58.4608	50.36604
lf-Pt	6.539254	6.629584	6.239184
lf-PuA	182.463	175.5583	150.0718
lf-PuI	151.2756	154.531	138.726
lf-PuL	123.1327	134.7406	125.0957
lf-PuM	854.6671	779.0034	736.5568
lf-VA	364.8248	397.8157	340.5276
lf-VAmc	28.55659	31.07307	27.04921
lf-VLa	559.377	585.9731	534.2324
lf-VLp	699.2602	759.2448	672.0896
lf-VM	18.55997	19.64559	17.59458
lf-VPL	783.8474	844.3912	734.5783
lf-Whole_thalamus	5652.642	5622.447	4979.5
rh-AV	130.8434	107.5276	104.285
rh-CeM	62.1244	55.45355	48.06551
rh-CL	33.63634	28.65087	26.14143
rh-CM	213.33	221.675	208.5581
rh-LD	25.83505	18.26258	12.13563
rh-LGN	183.3781	164.859	134.9011
rh-LP	105.6465	95.88531	83.27562
rh-L-Sg	16.47282	18.80542	18.71431
rh-MDl	269.9493	253.1327	230.6535
rh-MDm	708.8589	633.1818	593.9285
rh-MGN	114.918	117.1189	102.668
rh-MV(Re)	11.02887	9.390319	6.728964
rh-Pc	3.374931	2.94308	2.593708
rh-Pf	48.73434	54.78222	47.45407
rh-Pt	5.996214	6.098988	5.437686
rh-PuA	208.8913	223.5418	193.5258
rh-PuI	182.626	203.4928	177.7739
rh-PuL	161.889	188.4466	163.0043
rh-PuM	956.5881	1002.704	898.1172
rh-VA	340.8498	370.3286	339.9549
rh-VAmc	29.64441	30.86262	28.17401
rh-VLa	541.2549	579.2112	519.2811
rh-VLp	700.2594	737.5595	657.7377
rh-VM	17.45699	19.59703	17.50687
rh-VPL	742.177	810.5024	704.2473
rh-Whole_thalamus	5813.964	5865.795	5178.104

**Table A4:** Medians of substructures from Amygdala (lh: left side, rh: right side).

Variable	CG Q2	SND Q2	SD Q2
lh-Lateral-nucleus	616.3684	608.9339	542.3933
lh-Basal-nucleus	419.4339	390.2282	337.067
lh-Accessory-Basal-nucleus	257.6557	236.9855	197.4864
lh-Anterior-amygdaloid-area-AAA	51.55009	50.54429	41.65633
lh-Central-nucleus	45.46808	40.86922	39.81099
lh-Medial-nucleus	25.89562	21.13942	17.60892
lh-Cortical-nucleus	25.32682	25.28271	21.66816
lh-Corticoamygdaloid-transitio	170.3703	155.4683	127.7356
lh-Paralaminar-nucleus	45.41394	44.19915	41.82177
lh-Whole_amygdala	1644.198	1562.221	1383.873
rh-Lateral-nucleus	633.5202	631.8963	572.0321
rh-Basal-nucleus	425.5786	399.0396	346.8798
rh-Accessory-Basal-nucleus	266.5776	246.6755	220.4747
rh-Anterior-amygdaloid-area-AAA	53.56824	50.89934	46.4302
rh-Central-nucleus	46.06809	43.14087	43.48341
rh-Medial-nucleus	28.1996	22.82126	25.00227
rh-Cortical-nucleus	27.63528	27.46292	24.37196
rh-Corticoamygdaloid-transitio	172.166	147.3925	141.5533
rh-Paralaminar-nucleus	46.9591	42.19772	41.0332
rh-Whole_amygdala	1717.07	1604.96	1452.154

## References

[1] Owen M.J., Sawa A., Mortensen P.B. (2016). Schizophrenia. Lancet.

[2] Green M.F., Hellemann G., Horan W.P., Lee J., Wynn J.K. (2012). From Perception to Functional Outcome in Schizophrenia: Modeling the Role of Ability and Motivation. Arch. Gen. Psychiatry.

[3] Urfer-Parnas A., Mortensen E.L., Parnas J. (2010). Core of Schizophrenia: Estrangement, Dementia or Neurocognitive Disorder?. Psychopathology.

[4] Stroup T.S., Olfson M., Huang C., Wall M.M., Goldberg T., Devanand D.P., Gerhard T. (2021). Age-Specific Prevalence and Incidence of Dementia Diagnoses Among Older US Adults with Schizophrenia. JAMA Psychiatry.

[5] Lin C.-E., Chung C.-H., Chen L.-F., Chi M.-J. (2018). Increased Risk of Dementia in Patients with Schizophrenia: A Population-Based Cohort Study in Taiwan. Eur. Psychiatry.

[6] Cai L., Huang J. (2018). Schizophrenia and Risk of Dementia: A Meta-Analysis Study. Neuropsychiatr. Dis. Treat..

[7] Herold C.J., Schmid L.A., Lässer M.M., Seidl U., Schröder J. (2017). Cognitive Performance in Patients with Chronic Schizophrenia Across the Lifespan. GeroPsych.

[8] Palmer B.W., Moore R.C., Eyler L.T., Pinto L.L., Saks E.R., Jeste D.V. (2018). Avoidance of Accelerated Aging in Schizophrenia?: Clinical and Biological Characterization of an Exceptionally High Functioning Individual. Schizophr. Res..

[9] Sheffield J.M., Karcher N.R., Barch D.M. (2018). Cognitive Deficits in Psychotic Disorders: A Lifespan Perspective. Neuropsychol. Rev..

[10] Thuaire F., Rondepierre F., Bacon E., Vallet G.T., Jalenques I., Izaute M. (2020). Executive Functions in Schizophrenia Aging: Differential Effects of Age within Specific Executive Functions. Cortex.

[11] Gauthier S., Loft H., Cummings J. (2008). Improvement in Behavioural Symptoms in Patients with Moderate to Severe Alzheimer’s Disease by Memantine: A Pooled Data Analysis. Int. J. Geriatr. Psychiatry.

[12] Lyketsos C.G., Peters M.E. (2015). Dementia in Patients with Schizophrenia: Evidence for Heterogeneity. JAMA Psychiatry.

[13] Harvey P.D. (2001). Cognitive Impairment in Elderly Patients with Schizophrenia: Age Related Changes. Int. J. Geriatr. Psychiatry.

[14] Davidson M., Harvey P., Welsh K.A., Powchik P., Putnam K.M., Mohs R.C. (1996). Cognitive Functioning in Late-Life Schizophrenia: A Comparison of Elderly Schizophrenic Patients and Patients with Alzheimer’s Disease. Am. J. Psychiatry.

[15] Rohde C., Agerbo E., Nielsen P.R. (2016). Does Schizophrenia in Offspring Increase the Risk of Developing Alzheimer’s Dementia. Dement. Geriatr. Cogn. Disord. Extra.

[16] Shah J.N., Qureshi S.U., Jawaid A., Schulz P.E. (2012). Is There Evidence for Late Cognitive Decline in Chronic Schizophrenia?. Psychiatr. Q..

[17] Okada N., Fukunaga M., Yamashita F., Koshiyama D., Yamamori H., Ohi K., Yasuda Y., Fujimoto M., Watanabe Y., Yahata N. (2016). Abnormal Asymmetries in Subcortical Brain Volume in Schizophrenia. Mol. Psychiatry.

[18] Shahab S., Mulsant B.H., Levesque M.L., Calarco N., Nazeri A., Wheeler A.L., Foussias G., Rajji T.K., Voineskos A.N. (2019). Brain Structure, Cognition, and Brain Age in Schizophrenia, Bipolar Disorder, and Healthy Controls. Neuropsychopharmacology.

[19] van Erp T.G.M., Hibar D.P., Rasmussen J.M., Glahn D.C., Pearlson G.D., Andreassen O.A., Agartz I., Westlye L.T., Haukvik U.K., Dale A.M. (2016). Subcortical Brain Volume Abnormalities in 2028 Individuals with Schizophrenia and 2540 Healthy Controls via the ENIGMA Consortium. Mol. Psychiatry.

[20] Narr K.L., Thompson P.M., Szeszko P., Robinson D., Jang S., Woods R.P., Kim S., Hayashi K.M., Asunction D., Toga A.W. (2004). Regional Specificity of Hippocampal Volume Reductions in First-Episode Schizophrenia. NeuroImage.

[21] Heckers S., Konradi C., Swerdlow N.R. (2010). Hippocampal Pathology in Schizophrenia. Behavioral Neurobiology of Schizophrenia and Its Treatment.

[22] Zheng F., Li C., Zhang D., Cui D., Wang Z., Qiu J. (2019). Study on the Sub-Regions Volume of Hippocampus and Amygdala in Schizophrenia. Quant. Imaging Med. Surg..

[23] Dorph-Petersen K.-A., Lewis D.A. (2017). Postmortem Structural Studies of the Thalamus in Schizophrenia. Schizophr. Res..

[24] Adriano F., Spoletini I., Caltagirone C., Spalletta G. (2010). Updated Meta-Analyses Reveal Thalamus Volume Reduction in Patients with First-Episode and Chronic Schizophrenia. Schizophr. Res..

[25] Dietsche B., Kircher T., Falkenberg I. (2017). Structural Brain Changes in Schizophrenia at Different Stages of the Illness: A Selective Review of Longitudinal Magnetic Resonance Imaging Studies. Aust. N. Z. J. Psychiatry.

[26] Harvey P.D., Rosenthal J.B. (2018). Cognitive and Functional Deficits in People with Schizophrenia: Evidence for Accelerated or Exaggerated Aging?. Schizophr. Res..

[27] Uwatoko T., Yoshizumi M., Miyata J., Ubukata S., Fujiwara H., Kawada R., Kubota M., Sasamoto A., Sugihara G., Aso T. (2015). Insular Gray Matter Volume and Objective Quality of Life in Schizophrenia. PLoS ONE.

[28] Yue Y., Kong L., Wang J., Li C., Tan L., Su H., Xu Y. (2016). Regional Abnormality of Grey Matter in Schizophrenia: Effect from the Illness or Treatment?. PLoS ONE.

[29] Zhou Y., Ma X., Wang D., Qin W., Zhu J., Zhuo C., Yu C. (2015). The Selective Impairment of Resting-State Functional Connectivity of the Lateral Subregion of the Frontal Pole in Schizophrenia. PLoS ONE.

[30] Zhuo C., Ma X., Qu H., Wang L., Jia F., Wang C. (2016). Schizophrenia Patients Demonstrate Both Inter-Voxel Level and Intra-Voxel Level White Matter Alterations. PLoS ONE.

[31] Russell A.J., Munro J.C., Jones P.B., Hemsley D.R., Murray R.M. (1997). Schizophrenia and the Myth of Intellectual Decline. Am. J. Psychiatry.

[32] Laks J., Fontenelle L.F., Chalita A., Mendlowicz M.V. (2006). Absence of Dementia in Late-Onset Schizophrenia: A One Year Follow-up of a Brazilian Case Series. Arq. Neuropsiquiatr..

[33] Rivas J., Libreros J., Trujillo M., Hurtado A., Camprodon J. (2020). Experimental Data on Demographic, Functional and Structures of Patients with Schizophrenia and Schizophrenia-Dementia. Data Brief.

[34] Hachinski V.C., Iliff L.D., Zilhka E., Du Boulay G.H., McAllister V.L., Marshall J., Russell R.W.R., Symon L. (1975). Cerebral Blood Flow in Dementia. Arch. Neurol..

[35] Yesavage J.A., Brink T.L., Rose T.L., Lum O., Huang V., Adey M., Leirer V.O. (1982). Development and Validation of a Geriatric Depression Screening Scale: A Preliminary Report. J. Psychiatr. Res..

[36] Brandt J. (1991). The Hopkins Verbal Learning Test: Development of a New Memory Test with Six Equivalent Forms. Clin. Neuropsychol..

[37] Osterrieth P.A. (1944). Le Test de Copie d’une Figure Complexe; Contribution à l’étude de La Perception et de La Mémoire. [Test of Copying a Complex Figure; Contribution to the Study of Perception and Memory]. Arch. Psychol..

[38] Buschke H., Fuld P.A. (1974). Evaluating Storage, Retention, and Retrieval in Disordered Memory and Learning. Neurology.

[39] Nicholas L.E., Brookshire R.H., Maclennan D.L., Schumacher J.G., Porrazzo S.A. (1989). Revised Administration and Scoring Procedures for the Boston Naming Test and Norms for Non-Brain-Damaged Adults. Aphasiology.

[40] Belleville S., Fouquet C., Hudon C., Zomahoun H.T.V., Croteau J. (2017). Consortium for the Early Identification of Alzheimer’s disease-Quebec Neuropsychological Measures That Predict Progression from Mild Cognitive Impairment to Alzheimer’s Type Dementia in Older Adults: A Systematic Review and Meta-Analysis. Neuropsychol. Rev..

[41] Dale A.M., Sereno M.I. (1993). Improved Localizadon of Cortical Activity by Combining EEG and MEG with MRI Cortical Surface Reconstruction: A Linear Approach. J. Cogn. Neurosci..

[42] Fischl B., Salat D.H., van der Kouwe A.J.W., Makris N., Ségonne F., Quinn B.T., Dale A.M. (2004). Sequence-Independent Segmentation of Magnetic Resonance Images. NeuroImage.

[43] Fischl B., Sereno M.I., Dale A.M. (1999). Cortical Surface-Based Analysis. NeuroImage.

[44] Fischl B., Sereno M.I., Tootell R.B.H., Dale A.M. (1999). High-Resolution Intersubject Averaging and a Coordinate System for the Cortical Surface. Hum. Brain Mapp..

[45] Han X., Jovicich J., Salat D., van der Kouwe A., Quinn B., Czanner S., Busa E., Pacheco J., Albert M., Killiany R. (2006). Reliability of MRI-Derived Measurements of Human Cerebral Cortical Thickness: The Effects of Field Strength, Scanner Upgrade and Manufacturer. NeuroImage.

[46] Reuter M., Rosas H.D., Fischl B. (2010). Highly Accurate Inverse Consistent Registration: A Robust Approach. NeuroImage.

[47] Sled J.G., Zijdenbos A.P., Evans A.C. (1998). A Nonparametric Method for Automatic Correction of Intensity Nonuniformity in MRI Data. IEEE Trans. Med. Imaging.

[48] Fischl B., Liu A., Dale A.M. (2001). Automated Manifold Surgery: Constructing Geometrically Accurate and Topologically Correct Models of the Human Cerebral Cortex. IEEE Trans. Med. Imaging.

[49] Fischl B., Dale A.M. (2000). Measuring the Thickness of the Human Cerebral Cortex from Magnetic Resonance Images. Proc. Natl. Acad. Sci. USA.

[50] Reuter M., Schmansky N.J., Rosas H.D., Fischl B. (2012). Within-Subject Template Estimation for Unbiased Longitudinal Image Analysis. NeuroImage.

[51] Iglesias J.E., Augustinack J.C., Nguyen K., Player C.M., Player A., Wright M., Roy N., Frosch M.P., McKee A.C., Wald L.L. (2015). A Computational Atlas of the Hippocampal Formation Using Ex Vivo, Ultra-High Resolution MRI: Application to Adaptive Segmentation of in Vivo MRI. NeuroImage.

[52] Iglesias J.E., Insausti R., Lerma-Usabiaga G., Bocchetta M., Van Leemput K., Greve D.N., van der Kouwe A., Fischl B., Caballero-Gaudes C., Paz-Alonso P.M. (2018). A Probabilistic Atlas of the Human Thalamic Nuclei Combining Ex Vivo MRI and Histology. NeuroImage.

[53] Aylward E., Walker E., Bettes B. (1984). Intelligence in Schizophrenia: Meta-Analysis of the Research. Schizophr. Bull..

[54] Bilder R.M., Lipschutz-Broch L., Reiter G., Geisler S.H., Mayerhoff D.I., Lieberman J.A. (1992). Intellectual Deficits in First-Episode Schizophrenia: Evidence for Progressive Deterioration. Schizophr. Bull..

[55] Caspi A., Reichenberg A., Weiser M., Rabinowitz J., Kaplan Z., Knobler H., Davidson-Sagi N., Davidson M. (2003). Cognitive Performance in Schizophrenia Patients Assessed before and Following the First Psychotic Episode. Schizophr. Res..

[56] Duan X., He C., Ou J., Wang R., Xiao J., Li L., Wu R., Zhang Y., Zhao J., Chen H. (2021). Reduced Hippocampal Volume and Its Relationship with Verbal Memory and Negative Symptoms in Treatment-Naive First-Episode Adolescent-Onset Schizophrenia. Schizophr. Bull..

[57] Heaton R.K., Gladsjo J.A., Palmer B.W., Kuck J., Marcotte T.D., Jeste D.V. (2001). Stability and Course of Neuropsychological Deficits in Schizophrenia. Arch. Gen. Psychiatry.

[58] Heinrichs R.W., Zakzanis K.K. (1998). Neurocognitive Deficit in Schizophrenia: A Quantitative Review of the Evidence. Neuropsychology.

[59] Hoff A.L., Svetina C., Shields G., Stewart J., DeLisi L.E. (2005). Ten Year Longitudinal Study of Neuropsychological Functioning Subsequent to a First Episode of Schizophrenia. Schizophr. Res..

[60] Hyde T.M., Nawroz S., Goldberg T.E., Bigelow L.B., Strong D., Ostrem J.L., Weinberger D.R., Kleinman J.E. (1994). Is There Cognitive Decline in Schizophrenia?: A Cross-Sectional Study. Br. J. Psychiatry.

[61] Kremen W.S., Buka S.L., Seidman L.J., Goldstein J.M., Koren D., Tsuang M.T. (1998). IQ Decline During Childhood and Adult Psychotic Symptoms in a Community Sample: A 19-Year Longitudinal Study. Am. J. Psychiatry.

[62] Kurtz M. (2005). Neurocognitive Impairment across the Lifespan in Schizophrenia: An Update. Schizophr. Res..

[63] Mockler D., Riordan J., Sharma T. (1997). Memory and Intellectual Deficits Do Not Decline with Age in Schizophrenia. Schizophr. Res..

[64] Palmer B.W., Dawes S.E., Heaton R.K. (2009). What Do We Know About Neuropsychological Aspects of Schizophrenia?. Neuropsychol. Rev..

[65] Reichenberg A., Weiser M., Rapp M.A., Rabinowitz J., Caspi A., Schmeidler J., Knobler H.Y., Lubin G., Nahon D., Harvey P.D. (2005). Elaboration on Premorbid Intellectual Performance in Schizophrenia: Premorbid Intellectual Decline and Risk for Schizophrenia. Arch. Gen. Psychiatry.

[66] Rund B.R., Melle I., Friis S., Larsen T.K., Midbøe L.J., Opjordsmoen S., Simonsen E., Vaglum P., McGlashan T. (2004). Neurocognitive Dysfunction in First-Episode Psychosis: Correlates with Symptoms, Premorbid Adjustment, and Duration of Untreated Psychosis. Am. J. Psychiatry.

[67] Seidman L.J., Buka S.L., Goldstein J.M., Tsuang M.T. (2006). Intellectual Decline in Schizophrenia: Evidence from a Prospective Birth Cohort 28 Year Follow-up Study. J. Clin. Exp. Neuropsychol..

[68] Sheitman B.B., Murray M.G., Snyder J.A., Silva S., Goldman R., Chakos M., Volavka J., Lieberman J.A. (2000). IQ Scores of Treatment-Resistant Schizophrenia Patients before and after the Onset of the Illness. Schizophr. Res..

[69] van Winkel R., Myin-Germeys I., Delespaul P., Peuskens J., De Hert M., van Os J. (2006). Premorbid IQ as a Predictor for the Course of IQ in First Onset Patients with Schizophrenia: A 10-Year Follow-up Study. Schizophr. Res..

[70] Weickert T.W., Goldberg T.E., Gold J.M., Bigelow L.B., Egan M.F., Weinberger D.R. (2000). Cognitive Impairments in Patients with Schizophrenia Displaying Preserved and Compromised Intellect. Arch. Gen. Psychiatry.

[71] Woodberry K.A., Giuliano A.J., Seidman L.J. (2008). Premorbid IQ in Schizophrenia: A Meta-Analytic Review. Am. J. Psychiatry.

[72] Casanova M.F., Rothberg B. (2002). Shape Distortion of the Hippocampus: A Possible Explanation of the Pyramidal Cell Disarray Reported in Schizophrenia. Schizophr. Res..

[73] Small S.A., Schobel S.A., Buxton R.B., Witter M.P., Barnes C.A. (2011). A Pathophysiological Framework of Hippocampal Dysfunction in Ageing and Disease. Nat. Rev. Neurosci..

[74] Byne W., Buchsbaum M.S., Kemether E., Hazlett E.A., Shinwari A., Mitropoulou V., Siever L.J. (2001). Magnetic Resonance Imaging of the Thalamic Mediodorsal Nucleus and Pulvinar in Schizophrenia and Schizotypal Personality Disorder. Arch. Gen. Psychiatry.

[75] Kemether E.M., Buchsbaum M.S., Byne W., Hazlett E.A., Haznedar M., Brickman A.M., Platholi J., Bloom R. (2003). Magnetic Resonance Imaging of Mediodorsal, Pulvinar, and Centromedian Nuclei of the Thalamus in Patients with Schizophrenia. Arch. Gen. Psychiatry.

[76] Sim K., Cullen T., Ongur D., Heckers S. (2006). Testing Models of Thalamic Dysfunction in Schizophrenia Using Neuroimaging. J. Neural Transm..

[77] Andreasen N.C., Paradiso S., O’Leary D.S. (1998). “Cognitive Dysmetria” as an Integrative Theory of Schizophrenia: A Dysfunction in Cortical-Subcortical-Cerebellar Circuitry?. Schizophr. Bull..

[78] Coscia D.M., Narr K.L., Robinson D.G., Hamilton L.S., Sevy S., Burdick K.E., Gunduz-Bruce H., McCormack J., Bilder R.M., Szeszko P.R. (2009). Volumetric and Shape Analysis of the Thalamus in First-Episode Schizophrenia. Hum. Brain Mapp..

[79] Phelps E.A. (2004). Human Emotion and Memory: Interactions of the Amygdala and Hippocampal Complex. Curr. Opin. Neurobiol..

[80] Killgore W.D.S., Rosso I.M., Gruber S.A., Yurgelun-Todd D.A. (2009). Amygdala Volume and Verbal Memory Performance in Schizophrenia and Bipolar Disorder. Cogn. Behav. Neurol..

[81] Ho N.F., Chong P.L.H., Lee D.R., Chew Q.H., Chen G., Sim K. (2019). The Amygdala in Schizophrenia and Bipolar Disorder: A Synthesis of Structural MRI, Diffusion Tensor Imaging, and Resting-State Functional Connectivity Findings. Harv. Rev. Psychiatry.

[82] Rivas J.C., Libreros J., Trujillo M. (2020). Control—Schizophrenia—Schizophrenia-Dementia. Zenodo.

